# The Effect of Magnesium on Reperfusion Arrhythmias in STEMI Patients, Treated With PPCI. A Systematic Review With a Meta-Analysis and Trial Sequential Analysis

**DOI:** 10.3389/fcvm.2020.608193

**Published:** 2021-01-11

**Authors:** Laszlo B. Szapary, Zsolt Szakacs, Nelli Farkas, Kristof Schonfeld, Dora Babocsay, Mate Gajer, Balint Kittka, Balazs Magyari, Peter Hegyi, Istvan Szokodi, Ivan G. Horvath

**Affiliations:** ^1^Institute for Translational Medicine, Medical School, University of Pécs, Pécs, Hungary; ^2^Szentágothai Research Centre, University of Pécs, Pécs, Hungary; ^3^Medical School, Heart Institute, University of Pécs, Pécs, Hungary

**Keywords:** PCI, percutaneous coronary intervention, magnesium, STEMI, reperfusion arrhythmia

## Abstract

**Aims:** The restoration of coronary circulation plays a crucial role in treating ST-segment elevation myocardial infarction (STEMI), however successful reperfusion with primary percutaneous coronary intervention (PPCI) may induce life-threatening arrhythmias. The relation between myocardial electrical instability, as a background factor in reperfusion arrhythmia, and magnesium administered periprocedurally is still questionable. Several randomized clinical trials have been conducted predominantly in the thrombolysis era. Due to the contradictory results of these studies, there is little evidence of the potential preventive effect of magnesium on reperfusion arrhythmias. The aim of our study is to review and meta-analytically analyze data from all studies published so far in the PPCI era, comparing STEMI patients who have undergone primary PCI and received either magnesium or a placebo before the reperfusion procedure.

**Methods and Results:** Our meta-analysis follows the points in the PRISMA protocol and, meets all of their criteria. We conducted a search in five scientific databases using the following keyword combination: (myocardial infarction OR myocardial injury OR acute coronary syndrome OR acs OR stemi) AND magnesium. The 7,295 collected publications were filtered with the Endnote program by title, abstract and full-text based on predefined criteria. A statistical analysis was performed on three randomized-controlled trials using three common parameters, involving 336 patients Trial sequential analysis (TSA) was applied to assess the risk of random error associated with sparse data and multiple testing which can affect cumulative meta-analysis. The incidence of ventricular tachycardias (VTs) was not significantly increased in the non-magnesium control group. (OR: 1.36; CI: 0.619; −2.986, *P* = 0.263). For the ejection fraction (EF), a non-significant decrease was observed in the magnesium group by weighted mean difference calculation. (WMD: 7.262, 95% CI: −0.238; 0.053; *P* = 0.057). There was significant decrease in the infarct zone wall motion index (IZWMSI) in the magnesium treatment group. (WMD: 0.384, 95% CI: −0.042; 0.811, *P* = 0.015). Based on the TSA assessments, the results of all parameters are not significant, objectively demonstrating the lack of reasonable data pertaining to our question.

**Conclusions:** The preventive effect of magnesium on reperfusion arrhythmia associated with primary PCI can still be considered contradictory based on previous studies. In our study, we found, that magnesium is ineffective with a very weak evidence, due to the small number of patients and the biases of the included studies, and a well-designed clinical trial is needed in this area, based on the TSA.

## Introduction

The restoration of blood flow plays a crucial part-in the treatment of patients with ST-segment elevation myocardial infarction (STEMI), however reperfusion can act as a double-edged-sword. Reperfusion injury could manifest in different modes of cell death (e.g., necrosis and apoptosis), microvascular dysfunctions (e.g., impaired vasomotion, microvascular obstruction, and intramyocardial hemorrhage), and malignant ventricular arrhythmias [e.g., ventricular tachycardia (VT), ventricular fibrillation (VF)]. These clinical complications play an important role in postinfarction mortality (1).

Reperfusion induced VT or VF is defined as arrhythmia occurring during the restoration of coronary circulation. It is a relatively frequent condition among STEMI patients, who have undergone primary percutaneous coronary intervention (PPCI), 4–5% of them being affected (2). However, in the OACIS study, a much higher incidence was reported, with 23% of patients who received PCI suffering from reperfusion-related ventricular arrhythmia within 12 h of the onset of symptoms (3).

This phenomenon has long been known, especially in the era of thrombolysis, several studies have discussed the risk factors for acute reperfusion-related arrhythmias and the possible preventive solutions (e.g., beta-blockers and magnesium) in STEMI patients (4, 5).

Numerous angiographic and clinical parameters are known to predispose patients to reperfusion induced malignant arrhythmias associated with primary PCI, such as a culprit lesion in the left main artery, peak creatine kinase >3,000 IU/L, inferior wall STEMI, infarction due to dominant right coronary artery occlusion, pre-PCI thrombolysis, 0–1 TIMI flow and Killip class > 1 (1–3).

Reperfusion-induced ventricular arrhythmias strongly divide the medical community. There are contradictory data on long-term mortality, but most studies suggest that malignant ventricular arrhythmias associated with PCI do not increase mortality in the long term (2, 6). Nevertheless, numerous authors have shown that malignant arrhythmias associated with primary PCI significantly increase both in-hospital and 30-day mortality (7, 8).

Treatment of arrhythmias during primary PCI is consistent with the general management of VT or VF also accepted by the European Society of Cardiology (ESC) (9). Although the treatment algorithm is well-described, there is little data on the prevention of these conditions.

The pathomechanism is not fully understood, acute ischemia, anaerobic metabolism and acidosis, in the infarct-related area, presumably cause the activation of different types of potassium channels, responsible for repolarization, resulting in high potassium efflux from the cells. Furthermore, a catecholamine stress response caused by AMI through lipolysis, can lead to magnesium soap formation and resultant depletion. The intracellular magnesium loss caused by ischemia, leads indirectly to calcium influx and mitochondrial accumulation (10, 11). This cascade generates an electrolyte imbalance that triggers the electrical instability of the cell membrane (12, 13). Thus, restoring coronary blood flow is certainly beneficial for myocardial metabolism, but may further increase the risk of life-threatening arrhythmias due to pathophysiological abnormalities in membrane potential (14).

Magnesium is the second most important intracellular cation present with a concentration of 10–30 mM. Magnesium plays a key role in the synthesis and repair mechanisms of DNA, takes part-in numerous biochemical anabolic and catabolic processes, including protein synthesis and glycolysis since it is a cation, and is essential for the functioning of more than 800 enzymes (15). Furthermore, Mg^2+^ plays an important role in the regulation of many ion channels such as Na^+^, K^+^, and Ca^2+^ channels. It reduces the influx of potassium into the myocytes through the inhibition of late rectifier K^+^ channels. It can prolong both the atrial and ventricular refractory period, thus providing protection against proarrhythmic substrates. In the case of hypomagnesaemia, these protective mechanisms may be eliminated, which, in the case of a persistent condition, may contribute to the development of chronic cardiovascular diseases. In case of chronic magnesium deficiency, intacellular accumulation of Na^+^ and Ca^2+^ ions increase the risk of hypertension and coronary vasospasm. Through metabolic effects, Mg^2+^ deficiency increases the risk of type 2 diabetes or metabolic syndrome, because they are involved in the positive modulation of the GLUT-4 channel, enhancing insulin sensitivity (16–18).

In our case, perhaps the most important mechanism is, that Mg^2+^ can prevent intracellular Ca^2+^ accumulation, by inhibiting L-type Ca^2+^ channels, with a negative inotropic and anti-ischemic effect (1, 11, 19, 20), thus leading to the idea that Mg^2+^ supplementation can protect against the reperfusion-induced pathological ion cascade. Numerous experimental studies have shown that Mg^2+^ can inhibit the influx of Ca^2+^ into ischemic cells, thereby increasing the depolarization threshold of the cells and reducing their excitability (20, 21). Further, it can reduce vascular resistance, thus lessening the work of the heart (22).

The potential anti arrhythmic effect of magnesium, administered before opening the vessel is still debated in clinical practice.

In the 1990s and early 2000s, during the thrombolysis era, several studies investigated the magnesium effects in STEMI patients on the prevention of reperfusion induced arrhythmias, and did not find magnesium to be effective on the prevention of reperfusion arrhythmias, with the exception of the LIMIT-2 study (5). Given the more rapid vascular opening and flow growth associated with PCI, it is relevant to summarize this effect of magnesium in studies, conducted in the PPCI-era.

Primary PCI is now considered the gold standard of treatment for STEMI. There is limited data and evidence on the relationship between reperfusion arrhythmias and preventive magnesium administration during percutaneous coronary intervention. Therefore, our study aims to review the literature on the effects of magnesium on reperfusion arrhythmias in patients with STEMI undergoing primary PCI.

## Methods

The systematic-review theme is based on the Preferred Reporting Items for Systematic Reviews and Meta-Analysis (PRISMA) guideline (23), and meets all of its criteria. The PICO format was used: P(opulation)- patients with STEMI, I(ntervention)- patients undergoing magnesium treatment, C(ontrol)- patients who did not get magnesium, O(utcome): VT or VF, EF, IZWMSI, myocardial viability, mortality, hospitalization, etc.

### Search Strategy

The article search was carried out in five databases: Pubmed, Embase, Web of Science, the WHO Global Health Library and Cochrane from inception to 20 June 2019 using the recommendations in the PRISMA statement (23). Two investigators conducted a comprehensive search with a combination of the following keywords: (myocardial infarction OR myocardial injury OR acute coronary syndrome OR acs OR stemi) AND magnesium. References in the articles that were found were screened for additional suitable publications. Our search identified a total of 7,295 articles in Embase, PubMed, Cochrane, Web of Science and the WHO Global Health Library databases.

### Inclusion and Exclusion Criteria

Articles were selected if they involved a control group and a magnesium treatment group with patients suffering from STEMI, requiring primary PCI. The Zwolle Trial (24), the first study to investigate the efficacy of primary PCI in patients with AMI, was published in 1993, therefore, earlier publications were ignored. Conference abstracts were also included if they contained sufficient data. The language was not an exclusion criterion. Case reports, case series, duplicate reports, and non-human trials were excluded.

### Selection Process

Records were managed with the EndNote X7.4 software (Clarivate Analytics, Philadelphia, PA, USA) to remove duplicates. The right or seemingly right articles were selected step by step, first by reading the title, and then by reading the abstract and finally the full text. The selection process, as well as the search, was carried out by two colleagues, based on the PRISMA guideline.

### Data Extraction

Both numerical and textual data were collected on a 2016 Excel sheet (Office 365, Microsoft, Redmond, WA, USA). The following data were collected: author, date of publication, type of clinical trial, geographic location, number of subjects treated and controls, patient age, patient's history, infarction localization, baseline serum Mg (mg/dl), incidence of spontaneous recanalization, before and after echocardiographic parameters, such as the infarct zone wall motion score index (IZWMSI), coronarographic parameters including TIMI and Rentrop score, baseline hemodynamic parameters such as heart rate, blood pressure and left ventricular end-diastolic pressure, ejection fraction, cardiac output, drugs used during percutaneous transluminal coronary angioplasty (PTCA), magnesium dose, time of magnesium administration, creatine kinase peak (U/L), incidence of VT or VF, heart failure, cardiogenic shock, re-infarction, and deaths. The data were extracted by two colleagues independently and then confirmed by a third party.

### Statistical Analysis

We used meta-analytical calculation tools to observe pooled effect sizes for the three studies we used meta-analitical calculation tools. In the case of VT, the odds ratio (OR) with a 95% confidence interval (CI) was calculated. In the case of the EF and IZWMSI, a weighted mean difference was conducted with a 95% CI, where the weights are based on the number of items, because the studies used the same scale as these variables. The random effects model developed by DerSimonian and Laird (25) was applied in all calculation. Heterogeneity was determined with Cochrane's Q and the *I*^2^ statistics. Because of the small number of studies, the existence of publication bias was not investigated. The analysis was done with STATA software version 15.

In meta-analytical calculations, low-level methodological examinations and publication biases can lead to false *p*-values and thus to type 1 errors. To correct these distortions, the trial sequential analysis (TSA) method is used to compare studies with the same design. TSA is a randomized effect-based meta-analytical model to estimate the amount of data that can be used to make significant decisions about a given parameter. The relation between the cumulative Z curve and the trial sequential monitoring boundary shows the expressiveness of the results. If the cumulative Z curve crosses the trial sequential monitoring boundary and the cumulative sample size of the analysis reaches the required sample size, firm evidence can be observed. For the analysis we used the TSA software tool from the Copenhagen Trial Unit, Center for Clinical Intervention Research, Denmark (version 0.9 beta, www.ctu.dk/tsa).

The points on the Z curve all indicate such data numbers. If the Z curve reaches the conventional boundary, the result of the statistical analysis is significant. If the Z curve reaches the futility area the result is not-significant and no data is expected to affect this in the future. If the Z curve does not affect either, the result is not significant, however, this may change in the future, depending on new data. The latter statement applies to all our results.

### Quality Assessment

A risk of bias assessment was first performed at the individual study-level based on the Revised Cochrane risk-of-bias tool for randomized trials (RoB 2). From the individual studies, we chose the one with the highest risk of bias. Then we summarized overall RoB-assessment on the interventions at the comparison level with the same method.

### Assessing of the Grade of Evidence

The GRADE system was used to assess the strength of evidence of our results. GRADE stands for Grades of Recommendation Assessment, Development, and Evaluation (26). GRADEpro Guideline Development Tool [Software]. McMaster University, 2020 (developed by Evidence Prime, Inc.) was used to evaluate the level of evidence.

## Results

### Results of the Selection Process

**Figure 1:**
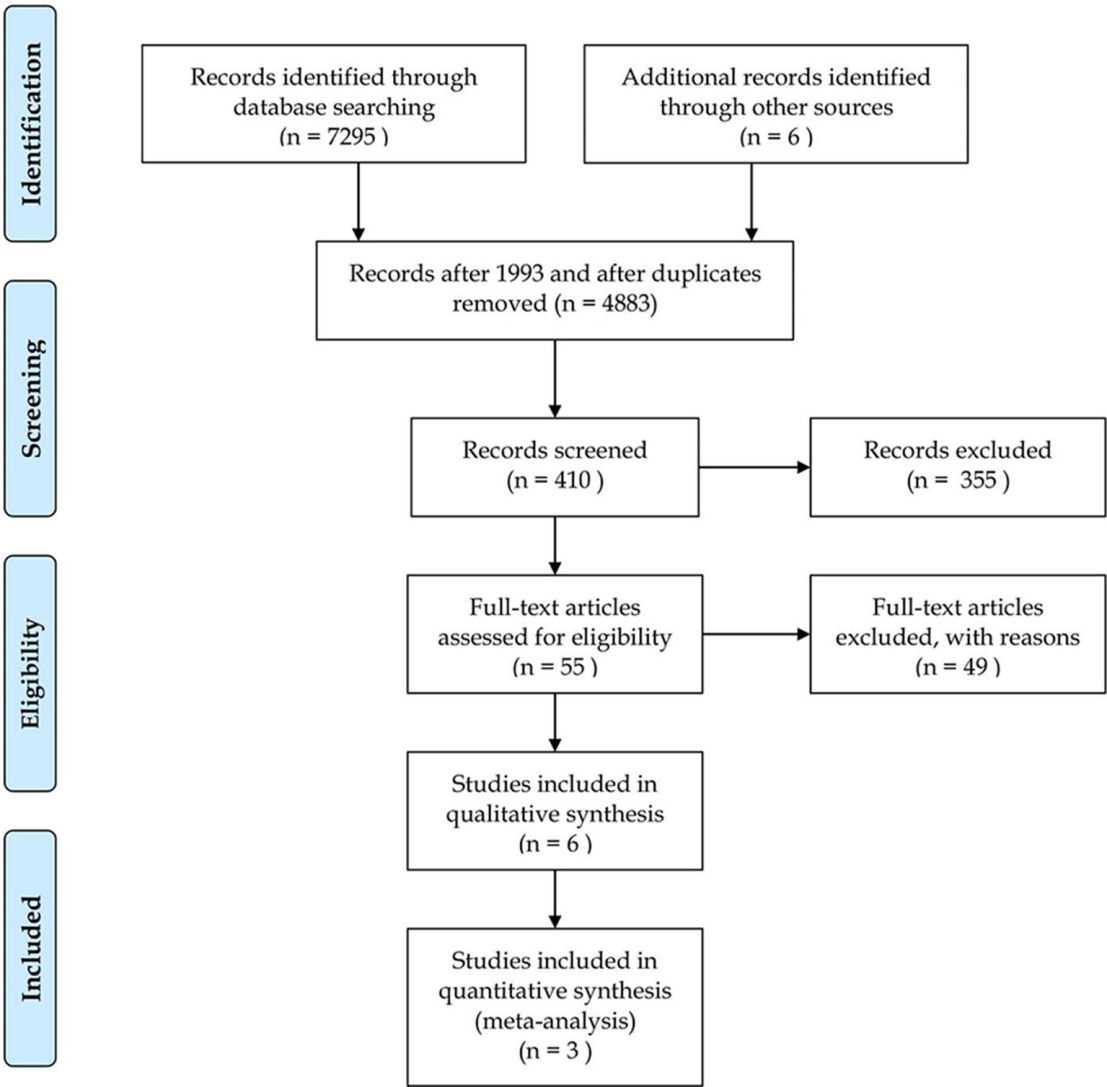
Preferred Reporting Items for Systematic Reviews and Meta-Analyses (PRISMA) study selection flow diagram.

Our search identified 4,127 articles in Embase, 1,621 in PubMed, 234 in the Cochrane database, 1,395 in Web of Science and 153 in the WHO Global Health Library. In the end three articles were eligible for the quantitative analysis with, 336 patients in total, 168 cases, and 168 controls. A further two articles provided results, but they were not suitable for meta-analytical calculations, as data from STEMI patients undergoing PCI or thrombolysis in these studies were aggregated and thus also excluded (27, 28). Due to the lack of publications, we attempted to save these articles and contact the authors via email to obtain the raw data, but were unsuccessful. Finally, we processed data from three articles (29–31) in more detail. The selection process is shown in the PRISMA flow chart, in Figure 1, and the main characteristics of the included studies are listed in Table 1.

**Table 1:** Characteristics of the included studies.

**Design**	**Country**	**Patients**		**Statistically analyzed outcomes**	**Statistically not analyzed outcomes**			**Follow-up**	**Comments**
					**Heart failure**	**Cardiogenic shock**	**Deaths**		
RCT	Italy	Mg group	75	VT, EF, IZWMSI	6	0	0	30 days	Patients with cardiogenic shock were excluded
		Placebo group	75		5	0	1		
RCT	Japan	Mg group	80	VT, EF, IZWMSI	18	10	1	21–28 days	After reperfusion, nicorandil infusion was started in both groups
		Placebo group	79		28	12	3		
RCT	Japan	Mg group	13	VT, EF, IZWMSI	N/A	N/A	N/A	28 days	After reperfusion, patients were maintained on 10,000–15,000 U/L heparin
		Placebo group	14		N/A	N/A	N/A		

### Results of the Statistical Analysis

The three articles were comparable for a total of three parameters, processing data from a total of 336 patients.

**Figure 2:**
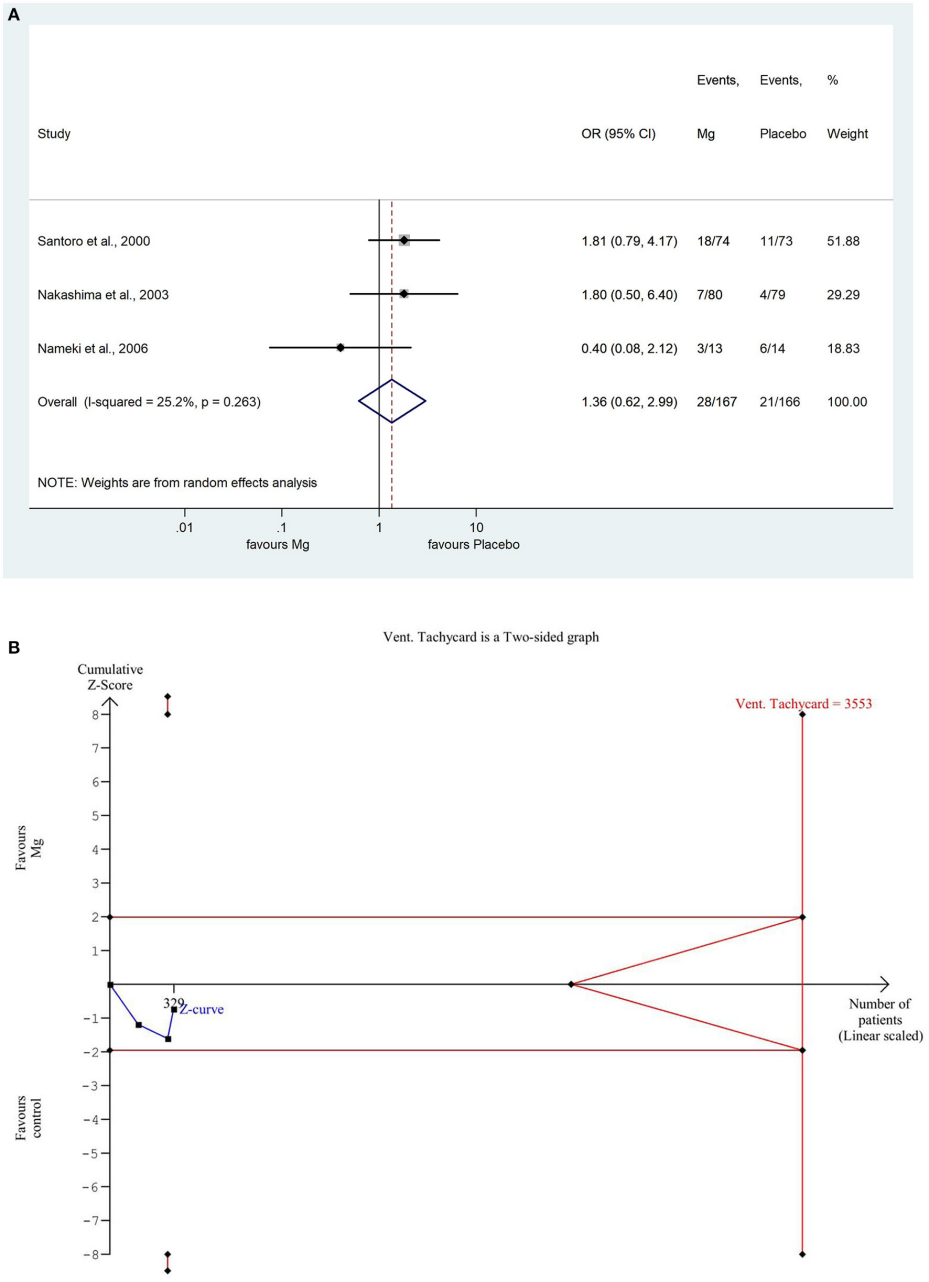
**(A)** Forest plot with the odds ratio method, of the 3 studies concerning the number of reperfusion associated ventricular tachycardias (VT) showing a non-significant increase in the magnesium group. **(B)** Trial sequential analysis shows that the number of VTs is non-significantly lower in the placebo group. More importantly, the Z curve does not intersect either the conventional boundary or the futility area, therefore, the result is not significant and may be strongly influenced by additional data in the future.

For VTs, we used the odds ratio method because this is an event number, so we arrive at the approximate number of times that a given event occurs. The pooled result showed that the number of VTs non-significantly increased in the magnesium treated group, using 95% confidence (OR: 1.36; CI: 0.619; 2.986, *P* = 0.263). There was a low degree of heterogeneity across the studies included in the analysis (*I*^2^: 25.2%; *P* = 0.263). A detailed result shown with the random effect model displayed in Figure 2A. Based on the TSA analysis, our result is not significant, however, this may change in the future with an adequate quantity of data (see Figure 2B).

**Figure 3:**
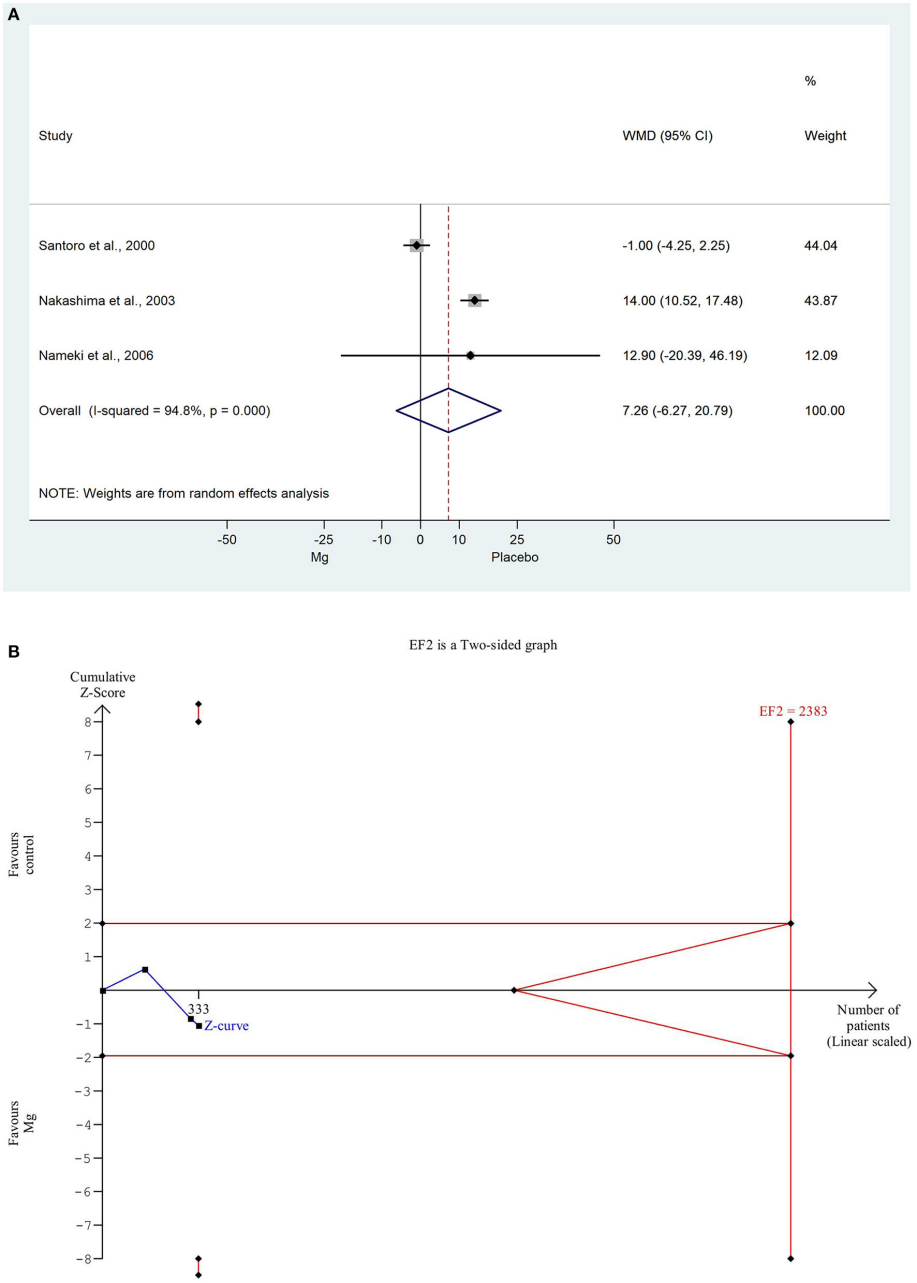
**(A)** Forest plot with weighted mean difference method, of the 3 studies concerning the ejection fraction (EF) during hospitalization, showing a non-significant decrease in the magnesium group. **(B)** Trial sequential analysis shows that EF is non-significantly decreased in the magnesium group. More importantly, the Z curve does not intersect either the conventional boundary or the futility area, therefore, the result is not significant and may be strongly influenced by additional data in the future.

The EF was comparable in all the three studies, which involved data on 336 patients, where employed the weighted mean difference method because the data used the same scale, so we were able to weight them based on the number of items. The weighted mean difference was non-significantly decreased in comparison with the placebo control group (WMD: 7.262, 95% CI: −0.238; −0.053; *P* = 0.057). There was a high degree of heterogeneity across the studies included in the analysis of the EF (*I*^2^: 94.8%, *P* = 0.000). A detailed result shown with the random effect model is presented in Figure 3A. Based on the TSA analysis, our result is not significant, however, this may change in the future with an adequate amount of data (see in Figure 3B).

**Figure 4:**
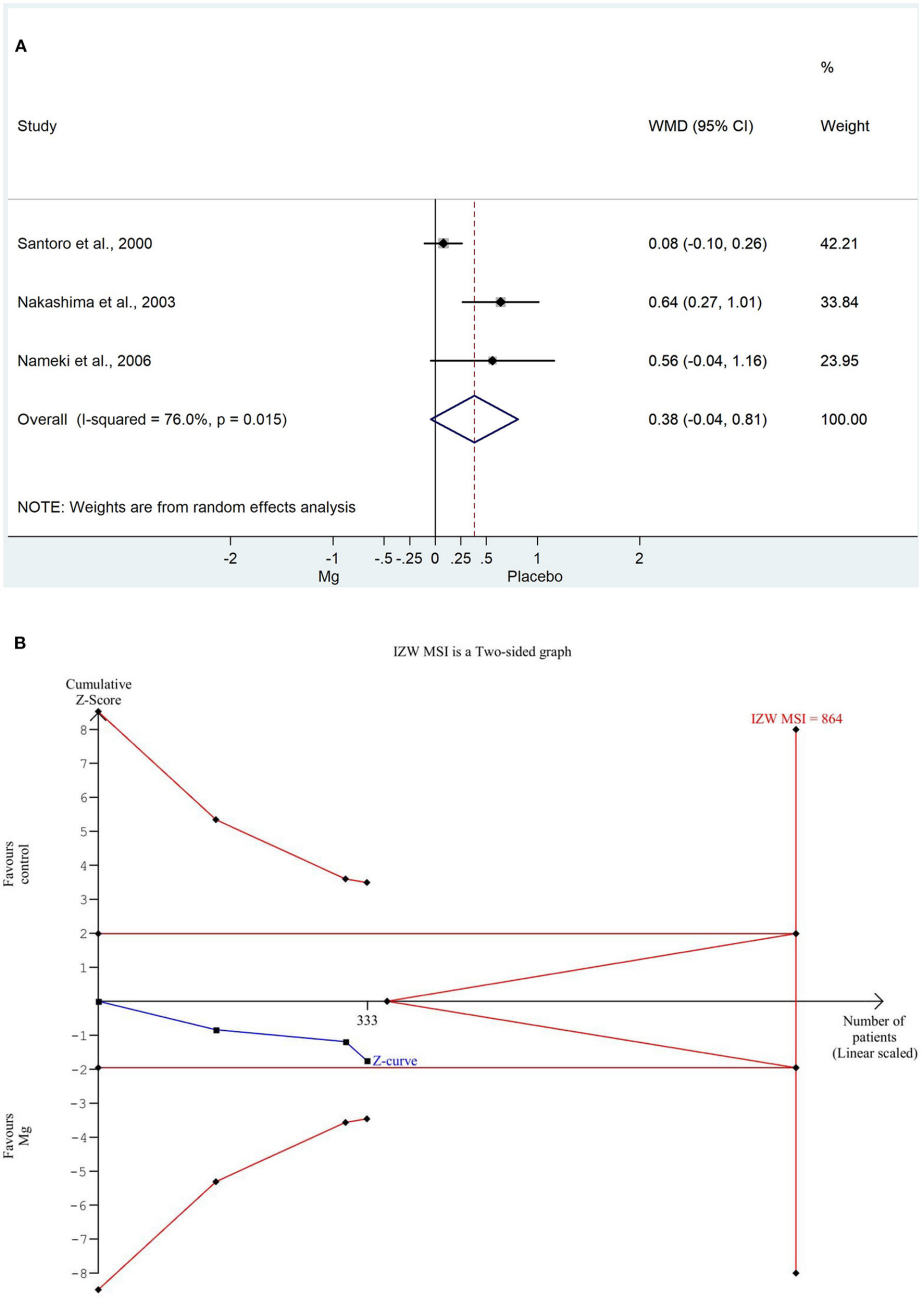
**(A)** Forest plot with weighted mean difference method, of the 3 studies concerning the infact zone wall motion severity index (IZWMSI) during the hospitalization, showing a significant decrease in the magnesium group. **(B)** Trial sequential analysis shows that IZWMSI, in contrast to the weighted mean difference method, is non-significantly decreased in the magnesium group. More importantly, the Z curve does not intersect either the conventional boundary or the futility area, therefore, the result is not significant and may be strongly influenced by additional data in the future.

For the IZWMSI, we used the weighted mean difference method again for the same reason. The weighted mean difference was significantly decreased in the magnesium group (WMD: 0.384, 95% CI: −0.042; −0.811, *P* = 0.015). There was a high degree of heterogeneity across the studies included in the analysis (*I*^2^: 76.0%; *P* = 0.015). A detailed result of the analysis shown with the random effect model is illustrated in Figure 4A. Based on the TSA analysis, our result is not significant, however, this may change in the future with an adequate amount of data (see in Figure 4B).

Two of the three articles (30, 32) stated other very important parameters, such as heart failure, cardiogenic shock, or death. Due to the low number of events of complications, a statistical analysis was not possible. These data are shown in Table 1, complete with the main characterisctics of the studies.

After performing the qualitative analysis (Rob2) and the GRADE method, all statistical results can be evaluated at a very low level of evidence.

The results of the statistical analysis are displayed in Table 2.

**Table 2:** Summary of findings.

**Outcomes**	**Results**	**Comments**	**Number of patients**	**Quality of evidence (GRADE)**
				
VT	OR: 1.3	Non-significantly increased in the placebo group	336	Very low
	95% CI: 0.619; −2.986 *P* = 0.263			
EF	WMD: 7.262	Non-significantly improved in the placebo group	336	Very low
	95% CI: −0.238; 0.053; *P* = 0.057			
IZWMSI	WMD: 0.384	Non-significantly improved in the placebo group	336	Very low
	95% CI: −0.042; 0.811, *P* = 0.015			

*VT, ventricular tachycardia; EF, ejection fraction; IZWMSI, infarct zone wall motion severity index; OR, odds ratio; WMD, weighted mean difference*.

Findings from the quality assessment of the randomized controlled trials using the RoB-2 scoring system are presented in Table 3.

**Table 3:** Revised Cochrane risk-of-bias tool for randomized trials (RoB 2).

	**Item 1**	**Item 2**	**Item 3**	**Item 4**	**Item 5**	**Overall**
Santoro et al. (29)	Yes	?	Yes	Yes	?	Low risk of bias
Nakashima et al. (31)	Yes	?	Yes	?	?	Intermediate risk of bias
Nameki et al. (30)	No	No	Yes	No	?	High risk of bias

*Item 1, Randomization process; Item 2, Deviations from intended interventions; Item 3, Missing outcome data; Item 4, Measurement of the outcome; Item 5, Selection of the reported result*.

## Discussion

Restoration of blood flow, in the occluded coronary artery plays a crucial role in the management of acute myocardial infarction. Nowadays, the gold standard treatment of STEMI is PCI. However, in some cases, opening the vessel can lead to life-threatening arrhythmias. The potential preventive effect of magnesium on reperfusion arrhythmias has been a topic of concern to date. Several studies and reviews have reported these positive effects (28, 32). Over the past decades, numerous clinical studies have been conducted to clarify the issue and yielded conflicting results. Possible contradictions may be due to reasons such as heterogeneity of the examined population, differences in hemodynamic instability in patients, dose of magnesium, timing of administration, and use of different reperfusion techniques.

Several meta-analyses have been conducted to investigate the association between non-PCI revascularization techniques induced arrhythmia and magnesium. In 1992, Horner et al. published a meta-analysis of eight studies, which contained data from 930 patients (33). All patients with acute myocardial infarction were revascularized with thrombolysis therapy. Their study showed that the periprocedural administration of magnesium significantly decreased ventricular and supraventricular reperfusion arrhythmias by 49 and 54%, respectively. The most recent meta-analysis in 2018 analyzed data from 22 articles and 6,061 patients (32). A major limitation of this study is that the data on patients treated with different revascularization techniques were pooled, thus potentially distorting the possible effects of magnesium. In any case, this meta-analysis also showed significant improvement in the magnesium group as regards the number of reperfusion arrhythmias. An interesting subgroup analysis was also performed in this analysis. In the magnesium group, patients who received magnesium ≥10 g, were separated, but higher efficacy was not demonstrated with higher doses of magnesium.

ISIS-4 (4) and LIMIT-2 (5), two monumental studies in the 1990-s, during the thrombolysis era, examined the effects of magnesium in patients with AMI, and produced conflicting results. ISIS-4 with 58,080 patients found no significant difference in 5-week mortality in the magnesium vs. placebo group. A major limitation of the study was that only stable patients were selected for the study, in contrast to the LIMIT-2 design, where there was no such exclusion criterion, which may be a major influencing factor as the control group had lower mortality in the former trial. Another possibility of bias in ISIS-4 is the use of inaccurate time windows. There was too much variance within the time of initiation of thrombolytic therapy, and, if magnesium was administered, this was 1–2 h after lysis therapy. This may also be the reason for this difference, as in the LIMIT-2 study involving 2,316 patients, a 24% relative reduction (*P* = 0.04, CI = −1; −43%) in in-hospital mortality was observed in the magnesium group (5).

Cardiac surgery, thrombolysis and PCI all have different effects on homeostasis. Previous meta-analyses (32, 33) ignored this distortion and worked with pooled data to investigate the potential preventive effects of magnesium on reperfusion arrhythmias. Therefore, the role of magnesium in PCI-related reperfusion arrhythmias remains questionable. Through the extensive literature research, we found five promising articles, three of which were suitable for statistical analysis (29–31). The other two (29, 30) were not used due to the pooled data problem, previously noted. One such study was the MAGIC trial (28), where 6,213 AMI patients were randomized for magnesium and placebo. In the former group, 19 g magnesium was administered in 24 h. Patients over 65 years of age were revascularized, of whom 63% were treated with thrombolysis. Notably, magnesium treatment did not reduce either the number of VT's or mortality. On the other hand, in 1999, Shibata et al. reported 36 patients, revascularized with PCI or thrombolysis, in whom significantly less reperfusion arrhythmia was observed in the magnesium group (27).

The results from a register analysis in the United States were reported in 2001 by Ziegelstein et al. (34). One thousand three hundred twenty-six patients with AMI who underwent revascularization by various techniques received periprocedural magnesium. Patients with CABG or PCI had increased mortality in the magnesium group. No difference was found in the thrombolysis group compared to the placebo group.

From the three studies included in our analysis, Santoro et al. randomized 150 patients with STEMI and treated with PPCI for their research and examined the effects of magnesium (29). The infarct zone wall motion severity index was designated as the primary endpoint. A major limitation of thise study is that patients with a severe condition or cardiogenic shock were not selected. They found magnesium ineffective in terms of myocardial damage and short-term clinical outcome.

Nakashima et al. published a randomized study of 180 patients in 2001 (31). All STEMI patients were treated with PPCI. Ninety-one patients were administered a placebo, 89 were treated with magnesium, and additionally, both groups received nicorandil at a dose of 4 mg/h for 3 days after the intervention. It was demonstrated that magnesium sulfate administered before coronary intervention had a beneficial effect on left ventricular and microvascular function in STEMI patients.

A third study with a small number of patients was also selected. STEMI patients undergoing PPCI were divided into three groups (30). There were 13 patients in the magnesium group, 14 in the placebo group and 13 in the nicorandil group. The results of the first two of these groups were examined. All patients received 10–15 IU heparin and only LAD-affected STEMI patients were randomized. In 2006, Nameki et al. found that periprocedural magnesium was not effective compared to the placebo.

At the endpoints of our meta-analysis, magnesium was ineffective compared to placebo-controlled groups in STEMI patients undergoing primary PCI. At the same time, the TSA statistical method, which is a strength of our research, has shown for each endpoint that these results are not definitive and may be strongly influenced by newer data in the future.

### Limitations

There are several limitations in our meta-analysis. In addition to the very few selected publications, a number of factors in all of the selected articles greatly weakens the value of the statistical analysis: the low number of patients, differences in hemodynamic stability among patient populations, heterogeneous data, varying doses of magnesium, and different definitions of VT.

### Conclusion

Taking all of this into account, our statistical analysis and extensive literature review suggest that periprocedural intravenously administered magnesium seems to be ineffective in STEMI patients undergoing a percutaneous coronary intervention, however this result can only be expressed with a very low grade of evidence. Based on the TSA, our research highlights the need for further, well-designed studies on the effects of magnesium, on PCI-associated reperfusion-induced arrhythmias.

## Data Availability Statement

The raw data supporting the conclusions of this article will be made available by the authors, without undue reservation.

## Author Contributions

LS, IH, and IS designed the research and the study concept and wrote the article. LS and KS performed the data extraction. NF analyzed and interpreted the data. LS, DB, and MG performed the quality assessment. BM, BK, PH, and ZS supervised the study. IH, IS, and PH conducted a critical revision of the manuscript for important intellectual content. All authors granted final approval of the version of the article to be published.

## Conflict of Interest

The authors declare that the research was conducted in the absence of any commercial or financial relationships that could be construed as a potential conflict of interest.

## Abbreviations

STEMI: ST-segment elevation myocardial infarction
PPCI: primary percutaneous coronary intervention
TSA: trial sequential analysis
PRISMA: Preferred Reporting Items for Systematic Reviews and Meta-Analysis
VT: ventricular tachycardia
EF: ejection fraction
IZWMSI: infarct zone wall motion index
WMD: weighted mean difference
OR: odds ratio.
