# Supplementary material for: The Effect of Magnesium on Reperfusion Arrhythmias in STEMI Patients, Treated With PPCI. A Systematic Review With a Meta-Analysis and Trial Sequential Analysis
Source: Front Cardiovasc Med. 2021 Jan 11;7:608193. doi: 10.3389/fcvm.2020.608193 (PMC7873853; doi:10.3389/fcvm.2020.608193)
Supplement: Supplementary file 2 [file Data_Sheet_1.PDF]

Certainty assessment							№ of patients		Effect		Certainty	Importance
**№ of studies**	**Study design**	**Risk of bias**	**Inconsistency**	**Indirectness**	**Imprecision**	**Other considerations** [e]	**Magnesium**	**Placebo**	**Relative (95% CI)**	**Absolute (95% CI)**		
**VT/VF**												
3	randomised trials	serious [a]	serious [b/1]	serious [c]	serious [d]	none	28/167 (16.8%)	21/166 (12.7%)	**OR 1.36** (0.62 to 2.99)	**38 more per 1 000** (from 44 fewer to 176 more)	⊕◯◯◯ VERY LOW	CRITICAL
**EF**												
3	randomised trials	serious [a]	serious [b/2]	serious [c]	serious [d]	none	167	166	-	MD **7.26 % lower** (-6.27 lower to 20.79 higher)	⊕◯◯◯ VERY LOW	IMPORTANT
**IZWMSI**												
3	randomised trials	serious [a]	serious [b/3]	serious [c]	serious [d]	none	167	166	-	MD **0.38 lower** (-0.04 lower to 0.81 higher)	⊕◯◯◯ VERY LOW	IMPORTANT

CI: Confidence interval; **OR:** Odds ratio; **MD:** Mean difference

Explanations

a. ROB Scoring System: Santoro 2001: Low risk of bias, Nakashima 2004: Intermediate risk of bias, Nameki 2006: High risk of bias

b/1: I2 =25,2%, P =0,263, b/2: I2 = 94,8%, P = 0,00, b/3: I2 = 76%, P = 0,015.

c. a number of factors in all of the selected articles greatly weakens the value of the statistical analysis: the low number of patients, differences in hemodynamic stability among patient populations, heterogeneous data, varying doses of magnesium, and different definitions of VT

d. Based on the TSA, our research highlights the need for further, well designed studies on the effects of magnesium, on PCI-associated reperfusion-induced arrhythmias

e: Due to the small number of studies and the small number of patients, publication bias could not be evaluated. No automatic downgrading was performed.
